# Rate and associated factors of non-retention of mother-baby pairs in HIV care in the elimination of mother-to-child transmission programme, Gulu-Uganda: a cohort study

**DOI:** 10.1186/s12913-017-1998-5

**Published:** 2017-01-18

**Authors:** Gerald Obai, Ruth Mubeezi, Fredrick Makumbi

**Affiliations:** 1 Department of Physiology, Faculty of Medicine, Gulu University, P.O. Box 166, Gulu, Uganda; 2 Department of Disease Control and Environmental Health, Makerere University School of Public Health, P.O Box 7072, Kampala, Uganda; 3 Department of Epidemiology and Biostatistics, Makerere University School of Public Health, P.O. Box 7072, Kampala, Uganda

**Keywords:** Mother-baby pairs, Non-retention, eMTCT, Gulu, Hoima, Hospitals, Uganda

## Abstract

**Background:**

Poor retention in HIV care of mother-baby pairs remains a public health challenge in the elimination of mother-to-child transmission (eMTCT) of HIV. We determined the rate of non-retention and time to non-retention of mother-baby pairs and associated factors in Gulu district, Northern Uganda.

**Methods:**

Mother-baby pairs enrolled into the eMTCT programme at Gulu Regional Referral Hospital (GRRH) and Lacor Hospital (LH) were retrospectively followed for 18 months. The primary outcomes were the rate of non-retention and time to non-retention of mother-baby pairs in HIV care. Data were abstracted from the antiretroviral treatment and early infant diagnosis (EID) registers, and mother/baby appointment books at the health facilities. Additional data on possible reasons for non-retention were obtained from cross-sectional interviews of mothers. Time to non-retention was calculated as the duration between enrolment of mother-baby pair into care and the date when the mother and/or baby missed a scheduled visit and did not return within 30 days. Factors associated with time to non-retention were assessed using Cox proportional hazards regression analysis. The measures of association were expressed as hazards ratio (HR) with 95% confidence intervals. Alpha was set at 0.05. The adjusted analysis includes variables with *p* <0.2 in the bivariable analysis or considered potential confounders. The Analysis used Stata version 12.

**Results:**

A total of 410 mother-baby pairs were enrolled in this study. Overall, non-retention by 18 month was 30.5%; higher at GRRH (34.7%) than LH (25.8%), *p* = 0.049. Non-retention was higher among pairs where the infant had no EID, adjusted (adj) HR = 5.81; 95% CI (2.55, 13.24), non-disclosure of mother’s HIV status, adj.HR = 1.86; 95% CI (1.22, 2.85), and lack of privacy during counselling session, adj.HR = 1.86; 95% CI (1.26, 2.85). Non-retention was about 60% lower [adj.HR = 0.43; 95% CI (0.20, 0.92)] among pairs where the mothers understood and appreciated the importance of adhering to all clinic appointments together with the baby.

**Conclusion:**

Nearly a third of mother-baby pairs are not retained in HIV care. Lack of EID services, poor quality service, non-disclosure of mother’s HIV status, and understanding the importance of adhering to all appointments together with the baby, were associated with time to non-retention.

## Background

Mother-to-child transmission (MTCT) of HIV remains the most common source of paediatric HIV infection, accounting for 95% of cases, of whom, 90% are in sub-Saharan Africa [1]. In the absence of any interventions, the risk of mother-to-child transmission may be up to 25% during pregnancy, labour, and delivery. There is an additional risk of 5-20% during the breastfeeding period, leading to an overall transmission rate of up to 45% [2]. In Uganda, there has been an increase in the prevalence of HIV from 6.4% in 2009 to 7.3% in 2011, with women being disproportionately affected (8.3%) as compared to men (6.1%) [3]. The situation is worse in Gulu where the prevalence is 10.3% [4]. As a result of the high prevalence of HIV among women, up to 6.5% of pregnant women in Uganda are HIV-infected [4]. In 2009 in Uganda, an estimated 149,661 children below the age of 15 years were living with HIV, of whom about 90% acquired the infection through MTCT [5].

In 2012, the Ugandan government embraced the call by the World Health Organisation to eliminate mother-to-child transmission (eMTCT) of HIV by 2015. To achieve the target of eMTCT, programmes were to follow-up and treat HIV-positive mothers together with their children until the 18^th^ month of the child’s life when the final HIV status of the infant is determined [6, 7]. However, in sub-Saharan Africa, up to 81% of mother-baby pairs are not retained in care six months after delivery [8, 9]. In Uganda, loss to follow-up (LTFU) of mother-baby pair has been reported to be 53.4% [10]. Literature shows that the majority of mother-baby pairs who are not retained in eMTCT programmes are due to LTFU and not death [11], suggesting that the infants remain alive, but at higher risk of acquisition of HIV from their mother than infants who remain in care. Non-retention, therefore, hinders programmes from maximally achieving the goals of eMTCT [12]. Non-retention of HIV-exposed infants also denies the infants who are infected, the opportunity for prompt diagnosis and treatment; yet paediatric HIV-associated morbidity and mortality can be prevented with early diagnosis and treatment [12].

While the risk of HIV transmission through breastfeeding continues for up to two years, few exposed infants are re-tested after testing at 4–6 weeks of life [13, 14], which only identifies those children infected in the uterus or during delivery [15]. Consequently, many HIV-exposed children remain with unknown HIV status.

This study set out to determine, the rate of non-retained pairs, the time to non-retention, and factors associated with time to non-retention, in the 18 months after birth for mother-baby pairs in eMTCT in HIV care programmes in Gulu district, Northern Uganda. The findings of this study will help to develop strategies aimed at improving retention of mother-baby pairs in care which may reduce the risk of MTCT of HIV during the postpartum period.

## Methods

### Study design, setting and population

This was a retrospective single cohort of HIV-positive mothers and their HIV-exposed babies born between January 2010 and December 2012 at either Lacor Hospital (LH) or Gulu Regional Referral Hospital (GRRH), both located in Gulu district, Northern Uganda. We also collected additional data on possible reasons for non-retention, in a cross-sectional manner. We identified some of the mothers at the hospital, while others were traced using telephone contacts or sketch maps in their hospital care cards, for face-to-face interviews. The study was conducted between June and August 2014. The entire cohort had at least 18 months of being at risk of experiencing non-retention by June 2014.

Lacor hospital and GRRH are the two largest hospitals in Acholi sub-region, serving patients from the entire sub-region and beyond. Gulu district is located at, 02 45 N, 32 00E, approximately 340 kilometres North of Uganda’s capital city, Kampala. The population of Gulu district is 443,733 [16].

Only mothers aged 15 years and above and who were permanent residents of the study area were eligible to take part in the study. Mothers aged 15–17 years are emancipated minors who were included in their own rights, as provided for in the Research Ethics Committee (REC) guidelines [17]. Mothers who had delivered from other hospitals but enrolled in either GRRH or LH and those who had transferred care to other facilities were excluded from the study so as to minimise data incompleteness but to also maintain study eligibility.

### Sample size and sampling procedure

**Fig. 1:**
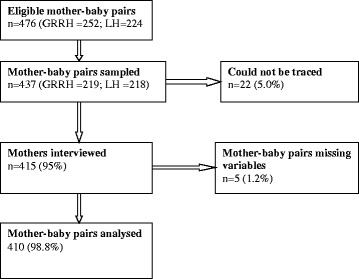
Diagram of mother-baby pairs recruitment into the study. A total of 476 mother-baby pairs were eligible for this study. We sampled 437 mother-baby pairs but 22 could not be traced for the face-to-face interviews. We therefore, collected data from 415 mother-baby pairs. However, we excluded five mother-baby pairs from the final analysis because they lacked or had unclear important variables like appointment dates missed, which were very important in determining non-retention in HIV care.

This study had two outcome variables, i) rate and ii) time to non-retention. We used the online StatsToDo software [18] to determine the sample size for the time to-non-retention, with 5% type-I error and power of 80%, giving a sample size of 393 mother-baby pairs. For the second outcome variable, rate non-retained within 18 months, a Kish Leslie [19] formula was used assuming a 53.4% rate of non-retention in Northern Uganda from a previous study [10], 5% margin of error. A 10% non-response rate was factored into the calculations. The largest sample size was considered for both outcomes because the study population was the same [20]. We sampled the study participants from the hospital ART registers. A total of 476 mother-baby pairs were eligible for the study (Fig. 1). Half of the study participants were recruited from each of the hospitals, and simple random sampling was conducted to enrol the study participants. The patient hospital number from the register was written on a piece of paper. The pieces of paper were put in a basin. We then randomly picked one at a time until the required sample size was attained. Probability sampling was used to avoid selection bias.

### Definition of variables

The variables for this study were derived from Andersen’s model of health care utilisation [21]. The outcome variables of this study were non-retention at 18 months, and time to non-retention of mother-baby pair in HIV care. Non-retention was defined as a mother and/or baby not returning at the facility within a period of 30 days after missing a scheduled visit. The rate of non-retention was calculated as the number of mother-baby pairs not retained in HIV care over the 18 month period divided by the total number of mother-baby pairs enrolled in the study. This was categorised as (“0” = retained and “1” = non-retained).

Time to non-retention was calculated as the duration from mother-baby pair enrolment into HIV care and when the mother and/or baby missed a scheduled visit and did not return for care within a period of 30 days. The unit of measurement was months. The period considered was from delivery to 18 months, thus entry into this study was from the date of birth. The event of interest was non-retention. Once the event of interest occurred, no re-entry in the study was allowed for this analysis.

Infant data collected included sex (male/female), feeding method (exclusive breastfeeding/mixed feeding); EID at 4–6 weeks (done/not done), and EID result (positive/negative). Maternal data collected included; age, categorised as 15–19, 20–29, ≥30 to reflect the health seeking behaviours among pregnant women in those categories, which also affect healthcare utilisation; marital status (categorised as never married, married, previously married), education (categorised on the basis of education levels in Uganda -none, primary, secondary, tertiary), religion (categorised on the basis of the main religious denominations in Uganda - Protestant, Catholic, Muslim, Pentecostal, plus the category Others, which incorporated religion or beliefs such as atheism, Hinduism, and Seventh Day Adventism, which are less widely practiced in Uganda). Occupation (categorised as housewife, farmer, wage/salaried workers, manual labour, others). This categorisation was on the basis of the widely recognised income earning sources of women in Uganda. Transport was categorised on the basis of the means of transport commonly used in Uganda (walk/bicycle/motorcycle/vehicle). Other maternal demographic data collected were residence (urban/rural), distance to the health facility (recoded as 0-5 km, 6-10 km, >10 km). Wealth index as determined by monthly income was categorised as [lowest = <UGX. 50,000 (~$15), second lowest = UGX. 50,000 - ≤100,000 ($15.1 - $30), middle = UGX. 100,000 - < 300,000 ($30.1 - $90), second highest = UGX. 300,000 - <500,000 ($90.1 - $151.5), highest = UGX. ≥ 500,000 (≥$151.5)]. The cut-off points for categorisation of wealth index were refined after pre-testing the study tools. Other data collected were the number of times delivered while HIV positive (1, 2+), partner disclosure (yes/no), spouse testing for HIV (yes/no), and the mother’s opinion about keeping all the hospital appointments (not important/important).

The quality of health care variables included privacy during counselling session (categorised as (yes/no), and waiting time, which was categorised as not considered long by the respondent versus considered long.

### Data collection

We abstracted data on HIV diagnosis, eMTCT enrolment, infant feeding, mother’s age, residence, marital status, distance to health facility, delivery, and follow-up outcomes from ART/EID registers, mother/baby appointment books kept at the health facilities, and exposed infant clinical charts. Records from the ART and EID registers and appointment books were used to determine the rate of non-retention of mother-baby pairs in care during the first 18 months of infant life. A structured questionnaire was developed to collect additional data on possible reasons for non-retention of mother-baby pair sampled from the clinic records. These were data not captured in the medical records but are considered important in understanding non-retention. The additional data included; the highest level of education attained, religion, occupation, means of transport to the hospital, wealth index, number of previous deliveries while HIV positive, whether the spouse had tested for HIV or not, and the mother’s opinion on the need to keep all appointments together with the baby, and the quality of care as determined by privacy and waiting time at the health facility. Some of the mothers were interviewed at the hospitals during their routine visits for care, while others were traced and interviewed from a place of their choice. The questionnaire and data abstraction form were pretested in a health facility setting. They were then refined to improve their validity and reliability. Trained research assistants with experience of working in HIV/AIDS units in hospitals collected data.

### Data management and analysis

Data were checked for completeness and accuracy at the end of each day of data collection. Double entry of data in an Excel spreadsheet was performed to detect inconsistent and missing entries. Data were then cleaned to eliminate errors in data entry and backed up regularly. We then exported data to Stata version 12 for statistical analysis.

We did survival analysis because it took into account the time to event (non-retention) information which is very important considering the long term and cascade nature of care in eMTCT of HIV. Data with missing outcome variables were excluded from the analysis. The missing data did not have any effect on our analysis since we had factored in a 10% non-response in the sample size calculations. In univariable analysis, summary statistics for categorical variables were analysed and presented as, proportions, frequencies, and graphs. Continuous variables are presented as range, mean, standard deviation (SD), and median. Kaplan–Meier methods were used to estimate the probabilities of non-retention of mother-baby pairs in the eMTCT programme by 18 months postpartum. In the bivariable analyses, the Log-Rank test was used to determine if there were any significant differences in the survival experiences between any two categories. Each categorical variable was assessed independently to test for association between the outcome variable (time to non-retention) and the independent variable. Cox proportional hazards regression analysis was used to determine independent association between mother-baby characteristics and time to non-retention, with variables that had *p* <0.20 at bivariable analyses or considered potential confounders being included in the multivariable analyses. We used hazards ratio as a measure of association.

## Results

### Mother-baby pair characteristics and main findings

A total of 410 mother-baby pairs took part in this study, providing a 95% response rate. Just over a half (52.7%) were from GRRH. The mothers were aged 15–44 years with a mean (SD) age of 28.6 (5.9) years. The majority of eligible participants were aged 20–29 years (53%), married (84.6%), and had attained some level of education (primary 38.1% or secondary 33.9%). Exclusive breastfeeding during the first six months was common (54.2%). EID was absent in 11 infants (2.7%), whilst 23 infants (5.8%) were detected as HIV positive by six weeks. Up to 83.2% of mothers disclosed their HIV-status (Table 1).

**Table 1:** Mother/baby characteristics

Parameter	Number	Percent
Baby’s sex		
Male	204	49.8
Female	206	50.2
Feeding method		
Exclusive breastfeeding	222	54.2
Mixed feeding	188	45.8
Early Infant Diagnosis (EID)		
Done	399	97.3
Not done	11	2.7
EID result		
Positive	23	5.8
Negative	376	94.2
Mother’s age		
15–19	20	4.9
20–29	220	53.7
≥ 30	170	41.4
Marital status		
Never married	23	5.6
Married	347	84.6
Previously married	40	9.8
Education		
None	70	17.1
Primary	156	38.1
Secondary	139	33.9
Tertiary	45	10.9
Religion		
Protestant	105	25.6
Catholic	227	55.4
Muslim	28	6.8
Pentecostal	36	8.8
Others	14	3.4
Occupation		
Housewife	125	30.5
Farmer	149	36.3
Wage/salaried workers	71	17.3
Manual labour	14	3.4
Others	51	12.5
Transport		
Walk	91	22.2
Bicycle	61	14.9
Motorcycle	167	40.7
Vehicle	91	22.2
Wealth index		
Lowest	125	30.5
Second lowest	128	31.2
Middle	104	25.4
Second highest	37	9.0
Highest	16	3.9
Residence		
Urban	259	63.2
Rural	151	36.8
Distance to health facility		
0–5 km	223	54.4
6–10 km	94	22.9
> 10 km	93	22.7
No. Of times delivered while HIV+		
1	214	52.2
2+	196	47.8
Partner disclosure		
Yes	341	83.2
No	69	16.8
Spouse tested		
Yes	316	77.1
No	94	22.9
Keeping all appointments with baby		
Not important	31	7.6
Important	379	92.4
Quality of counselling (privacy)		
Yes	316	77.1
No	94	22.9
Waiting time		
Not long	177	43.2
Long	233	56.8

**Fig. 2:**
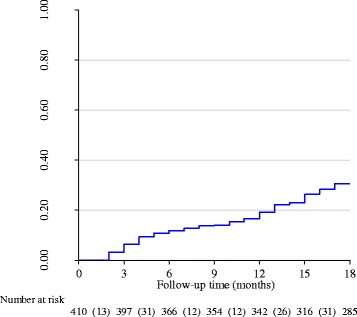
Non-retention over time

**Fig. 3:**
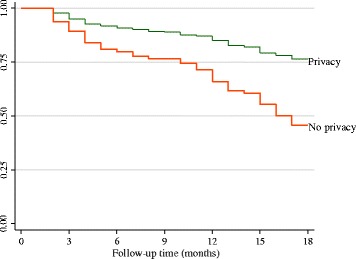
Plot of Kaplan-Meir for privacy

Nearly a third (30.5%) of the mother-baby pairs were not retained in care by end of month 18; higher in GRRH (34.7%) than in LH (25.8%), *p* = 0.049. The probability of non-retention was 0.12 at six months, 0.19 at twelve months and 0.3 at 18 months. The 18 month follow-up period was not long enough for the overall median time to non-retention to be ascertained (Fig. 2). It was calculated to be two months for babies with no EID, 15 months for mothers not disclosing their serostatus, while that for mothers who reported lack of privacy during counselling session it was 16 months (Fig. 3). The median time for those with EID, who disclosed their HIV serostatus, and those who reported that there was privacy during counselling could not be ascertained during the 18 month follow-up time.

Early infant diagnosis, education level attained, religion, occupation, transport, disclosure of HIV serostatus, the number of times delivered while HIV positive, spouse testing for HIV, quality of counselling, and waiting time were all significant factors for time to non-retention at the bivariable level (Table 2). Factors independently associated with time to non-retention were; absence of EID, adj. HR = 5.8; CI (2.55, 13.24), disclosing HIV status to a sexual partner adj. HR 1.86; CI (1.22, 2.85), and quality of counselling session adj. HR 1.86; CI (1.26, 2.85). Knowledge of the importance of adherence to all appointments together with the baby was associated with lower risk of non-retention adj. HR 0.43; CI (0.20, 0.92) (Table 3).

**Table 2:** Result of Log-Rank test of mother-baby non-retention in HIV care

Parameter	Not-retained/total	Percent not-retained	
	*n*	%	*P*-value
Baby’s sex			
Male	60/204	29.4	
Female	65/206	32.0	0.56
Feeding method			
Exclusive breastfeeding	63/222	28.4	
Mixed feeding	62/188	33	0.48
Early Infant diagnosis (EID)			
Done	115/399	28.8	
Not done	10/11	90.9	<0.001
EID result			
Positive	9/23	39.1	
Negative	106/376	28.1	0.24
Mother’s age			
15–19	4/20	20.0	
20–29	74/220	33.6	0.26
≥ 30	47/170	28.2	
Marital status			
Never married	8/23	34.8	
Married	101/347	29.1	0.44
Previously married	16/40	40.0	
Education			
None	36/70	51.4	
Primary	44/156	28.2	<0.001
Secondary	37/139	26.6	
Tertiary	8/45	17.8	
Religion			
Protestant	40/105	39.1	
Catholic	61/227	26.9	
Muslim	12/28	42.9	0.04
Pentecostal	11/36	30.6	
Others	1/14	7.1	
Occupation			
Housewife	54/125	43.2	
Farmer	36/149	24.2	
Wage/salaried workers	17/71	23.9	0.01
Manual labour	4/14	28.6	
Others	14/51	27.5	
Wealth index			
Lowest	45/125	36.0	
Second lowest	43/128	33.2	
Middle	24/104	23.1	0.18
Second highest	10/37	27.0	
Highest	3/16	18.8	
Residence			
Urban	84/259	32.4	
Rural	41/151	27.2	0.26
Distance from health facility			
0–5 km	72/223	32.3	
6–10 km	25/94	26.6	0.60
> 10 km	28/93	30.1	
Transport			
Walk	42/91	46.2	
Bicycle	20/61	32.8	
Motorcycle	46/167	27.5	0.001
Vehicle	17/91	18.7	
No. of times delivered while HIV+			
1	76/214	35.5	
2+	49/196	25.0	0.02
Partner disclosure			
Yes	86/341	25.2	
No	39/69	56.5	<0.001
Spouse tested			
Yes	80/316	25.3	
No	45/94	47.9	<0.001
Keeping all appointment with baby			
Not important	17/31	54.8	
Important	108/379	28.5	0.002
Quality of counselling (privacy)			
Yes	74/316	23.4	
No	51/94	54.3	<0.001
Waiting time			
Not long	40/177	22.6	
Long	85/233	36.5	0.002

**Table 3:** Unadjusted and adjusted HR for non-retention in HIV care by mother-baby characteristics

Parameter	Unadjusted	Adjusted HR	*P*-value
	HR (95% CI)	(95% CI)	
Baby’s sex			
Male	1.0		
Female	1.07 (0.75, 1.51)		
Feeding method			
EBF	1.0		
Mixed feeding	1.13 (0.80, 1.61)		
Early Infant Diagnosis (EID)			
Done	1.0	1.0	
Not done	13.19 (6.75, 25.77)	5.81 (2.55, 13.24)	<0.001
EID result			
Positive	1.0		
Negative	0.66 (0.33, 1.31)		
Mother’s age			
15–20	1.0		
20–29	1.83 (0.66, 5.00)		
≥ 30	1.51 (0.55, 4.20)		
Marital status			
Never married	1.0		
Married	0.81 (0.40, 1.67)		
Previously married	1.12 (0.48, 2.61)		
Education			
None	1.0		
Primary	0.49 (0.32, 0.77)		
Secondary	0.48 (0.31, 0.76)		
Tertiary	0.30 (0.14, 0.63)		
Occupation			
Housewife	1.0		
Farmer	0.51 (0.34, 0.78)		
Wage/salaried worker	0.50 (0.29, 0.86)		
Manual labour	0.60 (0.22, 1.65)		
Others	0.57 (0.32, 1.03)		
Religion			
Protestant	1.0		
Catholic	0.61 (0.41, 0.91)		
Muslim	1.13 (0.60, 2.15)		
Pentecostal	0.77 (0.40, 1.50)		
Others	0.15 (0.02, 1.08)		
Transport			
Walk	1.0		
Bicycle	0.77 (0.45, 1.30)		
Motorcycle	0.56 (0.37, 0.86)		
Vehicle	0.36 (0.21, 0.65)		
Distance to health facility			
0–5 km	1.0		
6–10 km	0.80 (0.50, 1.27)		
> 10 km	1.05 (0.68, 1.61)		
Wealth index			
Lowest	1.0		
Second lowest	0.89 (0.58, 1.34)		
Middle	0.59 (0.36, 0.97)		
Second highest	0.67 (0.34, 1.34)		
Highest	0.43 (0.14, 1.40)		
Residence			
Urban	1.0		
Rural	0.88 (0.61, 1.28)		
Delivery while HIV+			
1	1.0		
2+	0.70 (0.48, 1.00)		
Partner disclosure			
Yes	1.0	1.0	
No	2.83 (1.93, 4.13)	1.86 (1.22, 2.85)	0.04
Spouse tested			
Yes	1.0		
No	2.28 (1.58, 3.28)		
Keeping appointments			
Not important	1.0	1.0	
Important	0.46 (0.26, 0.84)	2.13 (0.82, 1.95)	0.17
Keeping baby appointment			
Not important	1.0	1.0	
Important	0.36 (0.22, 0.60)	0.43 (0.20, 0.92)	0.03
Privacy			
Yes	1.0	1.0	
No	2.90 (2.03, 4.14)	1.86 (1.26, 2.85)	0.01
Waiting time			
Not long	1.0	1.0	
Long	1.72 (1.18, 2.50)	1.32 (0.89, 1.95)	0.17

## Discussion

Nearly one-third of mother-baby pairs in this cohort were not retained in HIV care, which is less than previously reported in Northern Uganda [10]. However, if virtual elimination of mother-to-child transmission of HIV is to be achieved, then all the mother-baby pairs should be retained in HIV care. Studies have shown that non-retention affects continuous follow-up of HIV-exposed infants which allows early identification of infected infants and prompt initiation of treatment [22]. Non-retention also compromises the use of ART by the mother and baby which is important to prevent transmission of HIV during the breastfeeding period. The consequence for this is increased risk of MTCT of HIV.

Health facility factors such as; unavailable EID at 4–6 weeks, and poor quality counselling services as determined by lack of privacy during counselling session, and personal factors, such as; non-disclosure of mother’s HIV serostatus to the partner, and the mother’s knowledge of the importance of adherence to all appointments together with the baby were associated with time to non-retention in HIV care. Time to non-retention was shorter among mothers with babies who did not undergo EID at 4 to 6 weeks compared to those mother-baby pairs who had EID at 4 to 6 weeks postpartum, as recommended by WHO [23]. Failure to carry out EID could be due to shortages and interrupted supplies of materials, which have been reported to be contributing factors for non-retention [24]. When mothers do not receive services during follow-up visits, some studies have shown that they lose interest in the programme as they may feel that the health workers do not care about them [25]. This should be avoided as EID allows for early initiation of ART to improve survival in infants who are HIV-infected [26, 27]. Starting treatment early is important because when children are already severely immune-compromised, they fail to regain full immune function even after several years of treatment [28]. As a result, children who are initiated on ART late are more likely to die compared to when treatment is started early. Therefore, health departments should ensure constant availability of test kits for EID.

Mothers who felt that there was no privacy during counselling were at increased risk of non-retention. Their median duration in HIV care was 16 months. This finding is similar to that reported in Kenya [29]. Two possible explanations for lack of privacy are inadequate space or poor organisation of services within health facility, which compromises privacy and thus the confidentiality of mothers during counselling sessions [24]. Because of the stigma associated with HIV infection, and fear of involuntary disclosure of HIV status [25, 30], a mother who feels that her privacy is not being guaranteed may decide to opt out of long-term HIV care, which consequently increases the risk of maternal transmission of HIV through breastfeeding. Counselling rooms should ensure privacy so as to enable mothers to concentrate on the message being passed to them. This positive effect is emphasised in this study, which revealed approximately 60% lower risk of non-retention among pairs where the mothers understood and appreciated the importance of adhering to all clinic appointments together with their babies.

Studies have shown that non-disclosure remains a hindrance to prevention efforts against HIV in Africa [31]. In our study, non-disclosure of HIV positive serostatus to the spouse was significantly associated with time to non-retention. Similar findings have been reported in Malawi [32]. The main reason for non-disclosure is the stigma associated with it and yet there is evidence that disclosing one’s HIV status lessens the fear of accessing HIV care services [33] especially in our society where cultural norms place dominance on men with regards to women’s treatment decisions [34] as well as adherence to infant feeding advice [34].

Socio-economic status, of which education is a component, has been reported as one of the barriers to keeping women in HIV care [22, 35]. We report that women who had no formal education were at increased risk of non-retention. A similar finding has been reported in Kenya [36]. Maternal education has been reported to improve communication between a mother and healthcare provider as it enables mothers to retain information given to them during counselling sessions [37].

The strength of our study is that non-retention was defined as a mother-baby pair missing one scheduled appointment and not turning up at the HIV care point within 30 days of the missed scheduled appointment date. This is opposed to LTFU which considers missing three consecutive scheduled appointments, and yet missing even a single appointment, which affects the continuum of care, may increase the risk of maternal transmission of HIV.

### Study limitation

Data were primarily abstracted from routine care records, and so missed some important variables like education and transport. However, identification and follow-up of the participants were conducted to generate the missing variables. However, there could have been changes in some cases from the time the mothers were first enrolled into care.

## Conclusion

Non-retention of mother-baby pairs in HIV care remains high in Gulu district. Independent factors associated with time to non-retention were; lack of EID services, poor quality of counselling sessions, non-disclosure of mother’s HIV status, and understanding the importance of keeping all clinic appointments together with the baby. We, therefore, recommend that health facilities should ensure privacy during counselling sessions which would allow the mothers to remain focussed on the message being passed to them. Health workers should ensure that EID is done at the recommended time to prevent mothers from losing interest and opting out of eMTCT programme. Mothers should be encouraged during counselling sessions, to disclose their HIV serostatus to their spouse.

## Abbreviations

Adj. HR: Adjusted hazards ratio
ART: Antiretroviral treatment
CI: Confidence intervals
EID: Early infant diagnosis
eMTCT: Elimination of mother-to-child transmission
GRRH: Gulu Regional Referral Hospital
HIV: Human Immunodeficiency Virus
LH: Lacor hospital
UGX: Uganda shilling
WHO: World Health Organisation
